# The Relationship between Antibiotic Susceptibility and pH in the Case of Uropathogenic Bacteria

**DOI:** 10.3390/antibiotics10121431

**Published:** 2021-11-23

**Authors:** Annamária Kincses, Bálint Rácz, Zain Baaity, Orsolya Vásárhelyi, Erzsébet Kristóf, Ferenc Somogyvári, Gabriella Spengler

**Affiliations:** Albert Szent-Györgyi Health Center, Department of Medical Microbiology, Albert Szent-Györgyi Medical School, University of Szeged, Semmelweis utca 6, 6725 Szeged, Hungary; kincses.annamaria90@gmail.com (A.K.); racz.balint@med.u-szeged.hu (B.R.); baaity.zain@med.u-szeged.hu (Z.B.); orsolya.vasarhelyi.91@gmail.com (O.V.); krstf.erzsebet@gmail.com (E.K.)

**Keywords:** urinary tract infection (UTI), multidrug resistance, quorum sensing, efflux pump, biofilm, pH dependence

## Abstract

Urinary tract infections (UTIs) are common bacterial infections caused mainly by enteric bacteria. Numerous virulence factors assist bacteria in the colonization of the bladder. Bacterial efflux pumps also contribute to bacterial communication and to biofilm formation. In this study, the phenotypic and genetic antibiotic resistance of clinical UTI pathogens such as *Escherichia coli*, *Klebsiella pneumoniae*, and *Proteus mirabilis* were determined by disk diffusion method and polymerase chain reaction (PCR). Following this, different classes of antibiotics were evaluated for their antibacterial activity at pH 5, 6, 7 and 8 by a microdilution method. Gentamicin (GEN) was the most potent antibacterial agent against *E. coli* strains. The effect of GEN on the relative expression of *marR* and *sdiA* genes was evaluated by quantitative PCR. The slightly acidic pH (pH 6) and GEN treatment induced the upregulation of *marR* antibiotic resistance and *sdiA* QS activator genes in both *E. coli* strains. Consequently, bacteria had become more susceptible to GEN. It can be concluded that antibiotic activity is pH dependent and so the artificial manipulation of urinary pH can contribute to a more effective therapy of multidrug resistant bacterial infections.

## 1. Introduction

Urinary tract infections (UTIs) are one of the most frequently encountered bacterial infections in everyday health care. There are various classifications of UTIs: lower (limited to the bladder) or upper (pyelonephritis), complicated (in patients with a catheter, structural or functional abnormality or pregnant), or uncomplicated (none of the above). The diagnosis depends on the urinary symptoms and on the urine culture positive for an uropathogen exceeding a given threshold (which varies according to gender and the presence of a urinary catheter). Both patient and bacterial factors contribute to the development of UTIs. Anatomical or functional abnormalities, genetic predisposition, and certain behaviors (e.g., sexual intercourse) can increase susceptibility to uropathogens [1]. Several virulence factors aid the bacteria in the colonization of the bladder and the evasion of the immune system. These include the urease enzyme, adhesins, biofilm formation, toxins, and iron acquisition systems [2].

The dominant bacterial species involved in uncomplicated and complicated UTIs is uropathogenic *Escherichia coli* (UPEC). Other species include *Klebsiella pneumoniae*, *Staphylococcus saprophyticus*, *Enterococcus faecalis*, group B *Streptococcus* (GBS), *Proteus mirabilis*, *Pseudomonas aeruginosa*, *Staphylococcus aureus*, and *Candida* spp. [3]. Commonly used antibiotics for treating UTIs are trimethoprim sulfamethoxazole, third generation cephalosporins, ciprofloxacin, and ampicillin. However, as a result of emerging multidrug resistance, antibiotic treatment is becoming more difficult. This is more pronounced in the case of the Enterobacteriaceae family, which has resistance to third-generation cephalosporines and other antibiotics [4] through its production of extended spectrum β-lactamases (ESBL), class C β-lactamases (AmpC enzymes) and carbapenemases.

Emerging multidrug resistance and the high recurrence rate of UTIs pose a significant threat, particularly to women, infant boys, and older men. The use of prophylactic antibiotics is no longer sustainable. However, vaccine therapies and anti-virulence factor therapies could be promising strategies [5]. Another attractive approach to battle multidrug resistance is single-dose aminoglycoside therapy, saving carbapenems for treatment of severe infections. Aminoglycosides are excreted in high concentrations in urine and, with a single parenteral dose, patient non-adherence can be avoided [6,7].

Urinary pH levels vary broadly (pH 4.5–8) and can be easily modified by diet or medications. Modification of the urinary pH could play an important role both in the treatment and in the prevention of UTIs, since pH is an essential factor in the colonization and proliferation of uropathogenic bacteria and modifies the efficacy of antibiotics [8,9,10]. Siderocalin (SCN), a lipocain-type molecule produced also by uroepithelial cells, has a key function in the host defense system. It binds iron-binding siderophores (for example, enterobactin) released by microorganisms, however SCN activity levels can be influenced by pH and metabolites [11].

Quorum sensing (QS) is a cell–cell communication system which regulates gene expression based on population density. It enables bacteria to form biofilms and express various virulence factors, which can contribute to increased drug resistance [12]. QS is also involved in the pathogenesis of UTIs, especially catheter-associated urinary tract infections (CAUTIs). It regulates motility and biofilm formation, allowing the colonization of the bladder [13]. Urine composition is a crucial host factor that can alter the risk of a UTI; a pH less than 5, organic acids, and high urea content make the environment less ideal for bacterial growth. Moreover, urea in the urine is also able to inhibit the expression of QS related genes [14]. Efflux pumps (EPs) are membrane proteins that are mostly associated with antibiotic resistance, however they may also have a major role in the formation of biofilm and in QS regulation. EPs may take part in the efflux of antibiotics and metabolic intermediates, along with extracellular polymeric substances and QS molecules. They can influence aggregation and indirectly regulate biofilm-associated genes. Therefore, the development of molecules with efflux pump inhibitory activity may appeal in order to reverse multidrug resistance in bacteria and also as anti-biofilm agents [15].

## 2. Results

### 2.1. Antibiotic Susceptibility Test

In vitro antibiotic susceptibility tests were conducted on six UTI bacterial isolates and MIC breakpoints were determined according to EUCAST guidelines [16]. Fifteen antibacterial agents were used for the susceptibility testing (cefuroxime, ceftriaxone, ceftazidime, ceftazidime/avibactam, trimethoprim/sulfamethoxazole, ertapenem, imipenem, meropenem, gentamicin, tobramycin, amikacin, ciprofloxacin, norfloxacin, ampicillin, and amoxicillin-clavulanic acid).

*E. coli* 32313 was susceptible to all antibiotics but resistant to trimethoprim/sulfamethoxazole, gentamicin, ciprofloxacin, and norfloxacin. *E. coli* 33504 was completely susceptible to all antibiotics. *K. pneumoniae* 33443 was resistant to ampicillin, ciprofloxacin, and norfloxacin. *K. pneumoniae* 33163 was susceptible to ceftazidime/avibactam and intermediate for tobramycin and amikacin but was resistant to the other antibiotics. *P. mirabilis* 33877 was resistant to ampicillin and trimethoprim/sulfamethoxazole but was susceptible to all other tested antibiotics. *P. mirabilis* 32470 was susceptible to ceftazidime/avibactam and intermediate to amikacin but was resistant to the other antibiotics tested. All strains were susceptible to ertapenem, meropenem, and imipenem.

The results of phenotypic and genetic investigations are presented in Table 1 and Table 2, respectively.

### 2.2. Genetic Investigation

By PCR 3 *tem*, 2 *shv*, 1 *ctx-m* and 1 *oxa* amplicons were found for ESBL screening. Furthermore, 4 *oqxAB* and 3 *aac(69)-Ib-cr* and quinolone resistance genes were detected. In addition, 4 *sul2* and 1 *sul3* sulfonamide resistance genes were identified. The results of the genetic investigations are presented in Table 2.

### 2.3. Antibacterial Activity

After the determination of resistance genes, different classes of antibiotics, namely erythromycin (ERY), ampicillin (AMP), ciprofloxacin (CIP), and gentamicin (GEN), were evaluated for their antibacterial activity. Since urinary pH could have an impact on the treatment of UTIs, the activity of antibiotics at different pH values is a critical issue. This antibiotic evaluation was performed at pH 5, 6, 7 and 8 by microdilution method on clinical strains of *E. coli*, *P. mirabilis*, and *K. pneumoniae*. The results show that ERY had no antibacterial effect at pH 5 and 6. At pH 7 and 8, significant activity was observed on sensitive *E. coli* 33504 and *K. pneumoniae* 33443 strains. ERY prevented the growth of tested bacteria most effectively in alkaline environment (pH 8; Table 3).

AMP had no effect on the tested strains (MIC greater than 100 µg/mL) except *E. coli* 33504. Here, AMP showed potent antibacterial activity at pH 5–7 (MIC: 12.5 µg/mL; Table 3). pH dependence was also detected for CIP on *E. coli* 33504, *K. pneumoniae* 33443, *K. pneumoniae* 33163, and *P. mirabilis* 33877 by showing higher antibacterial activity at pH 7 and 8 (Table 3). GEN was the most active antibiotic at alkaline pH of all tested strains (Table 3).

### 2.4. Relative Expression of marR and sdiA Genes

**Figure 1:**
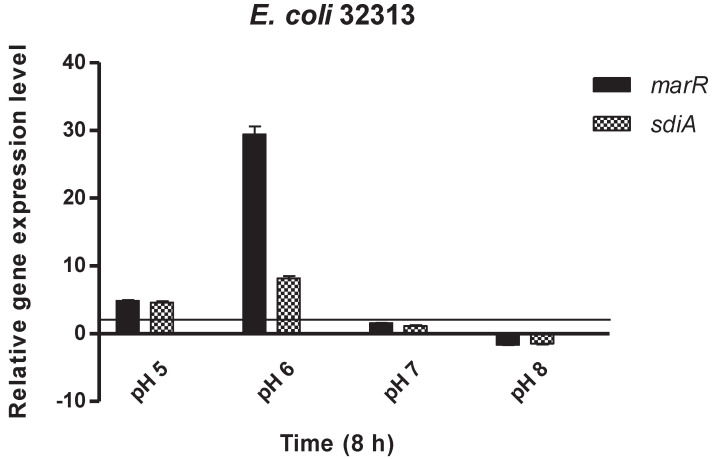
Relative gene expression levels of *marR* and *sdiA* genes in the presence of gentamicin in *Escherichia coli* 32313 after 8 h exposure. The line denotes the threshold value, which was set at a two-fold increase in transcripts.

**Figure 2:**
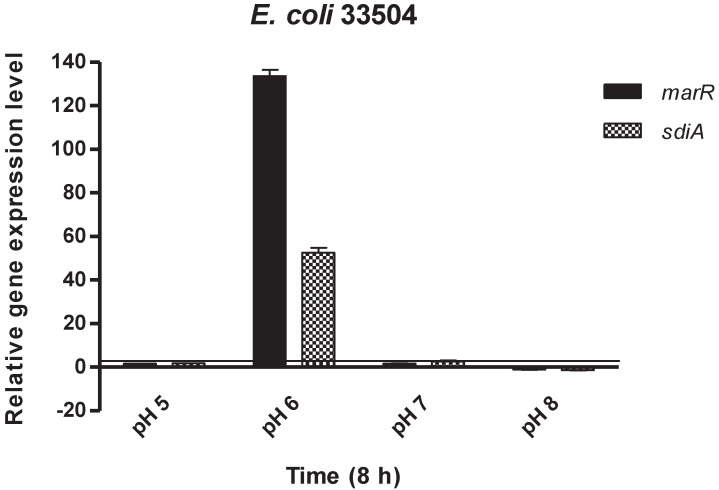
Relative gene expression levels of *marR* and *sdiA* genes in the presence of gentamicin in *Escherichia coli* 33504 after 8 h exposure. The line denotes the threshold value, which was set at a two-fold increase in transcripts.

GEN was the most potent antibacterial agent against *E. coli* strains (33504 and 32313) and, for this reason, the effect of GEN on the relative expression of *marR* and *sdiA* genes in both *E. coli* strains was evaluated. The *E. coli marR* gene encodes a repressor of the *marRAB* operon, a regulatory locus controlling multiple antibiotic resistance. In addition, *sdiA* encodes the transcription factor SdiA, a LuxR homolog that can respond to acyl-homoserine lactone (AHL), which, in turn, is related to quorum sensing. As shown by Figure 1 and Figure 2, GEN treatment in a pH 6 environment induced a significant stress response in both *E. coli* strains with the *marR* and *sdiA* genes being upregulated compared to the other pH levels. In contrast, *marR* and *sdiA* genes at pH 8 and in the presence of GEN were downregulated in the tested strains (Figure 1 and Figure 2).

## 3. Discussion

The results demonstrate that the genetic data agrees mostly with the phenotypical investigations, although there are some differences between the two methods. In *E. coli* 32313 clinical strain, having taken into consideration that the *aac(69)-Ib-cr* gene is also responsible for concurrent aminoglycoside and fluoroquinolone resistance induction [17], the genetic background of fluoroquinolone and the sulfonamide resistance agrees with the phenotype. *P. mirabilis* 33877 strain has one fluoroquinolone resistance gene present (*oqxAB*), which does not correspond to the phenotypic investigation. Finally, *K. pneumoniae* 33443 contained the *sul2* gene, which was phenotypically inactive.

There are two possible reasons for these differences. The first possible reason is that the genetic investigation was not quantitative and there were insufficient copies of the gene. The genetic investigation could be quantified using more sensitive quantitative real-time PCR. Additionally, *oqxAB* confers low to intermediate resistance to quinolones [16]. According to the literature, there is no complete agreement between phenotypical and genetic methods, therefore these differences could have been caused by multiple factors, for example, by the lack of promoter regions (an IS26 element in the case of *oqxAB*) [18,19]. It was demonstrated that the acidic pH and promethazine treatment induced a significant stress response in *E. coli*. Moreover, the genes *marB*, *marR*, *acrA*, *acrB*, *soxS*, *ftsI* and *sdiA* were up-regulated at an acidic pH compared to the treatment at a neutral pH [20].

In this study, the activity of the antibiotics of different classes was studied by broth microdilution method at pH 5, 6, 7 and 8 on sensitive and resistant UTI bacterial strains. It can be concluded that the activity of ERY, CIP, and GEN was more effective in an alkaline environment on the tested strains. Furthermore, AMP showed a more potent efficacy at acidic and neutral pH levels on *E. coli* 33504. Urine pH can be modified to prevent certain urological diseases [10]. Patient urinary pH can be acidified by ascorbic acid and ammonium chloride while becoming more alkaline with sodium bicarbonate or potassium citrate [10]. The results confirmed that the activity of antimicrobial drugs is pH dependent. This enables the artificial manipulation of urinary pH to contribute to a more effective therapy of urinary tract infections, especially in cases of infections caused by multidrug resistant bacteria. Additionally, this technique could reduce the cost of treatment.

The slightly acidic pH (pH 6) and GEN treatment induced the upregulation of *marR* antibiotic resistance and *sdiA* QS activator genes in both *E. coli* strains, increasing bacterial susceptibility. A possible explanation for this could be the pH-dependent activity of siderocalin (SCN) protein, which is produced by uroepithelium. This has the ability to bind the iron-binding enterobactin. In a previous study, the activity of SCN increased at pH > 6.45. The elevated pH facilitated host-derived ferric-aryl complex assembly in SCN, leading to the iron starvation of uropathogenic *E. coli* (UPEC) [11]. This observation suggests that *E. coli* at pH 6 is more susceptible to the antimicrobial agents that caused the over-expression of *marR* and *sdiA* genes. It needs to be highlighted that our study represents in vitro results lacking the response of the host to UPEC, therefore host factors should also be included in further in vivo studies.

## 4. Materials and Methods

### 4.1. Bacterial Strains

Clinical strains of *Escherichia coli* 33503, 32313; *Klebsiella pneumoniae* 33443, 33163; *Proteus mirabilis* 3387, 32470 were provided by the Institute of Clinical Microbiology at the University of Szeged and were included for the investigations. The species identities of the clinical isolates were confirmed by both MALDI-TOF MS and conventional biochemical methods.

### 4.2. Determination of Minimum Inhibitory Concentrations by Microdilution Method

The minimum inhibitory concentrations (MICs) of antibiotics (erythromycin (ERY), ampicillin (AMP), ciprofloxacin (CIP), and gentamicin (GEN)) were determined by microdilution method in 96-well plates according to the Clinical and Laboratory Standards Institute (CLSI) guidelines using MHB at pH 5, 6, 7 and 8 [21]. The bacterial strains were separately cultured in media of pH 5 to pH 8 overnight at 37 °C and the bacterial culture grown at the appropriate pH was applied in the assay.

### 4.3. Disk Diffusion

The antibiotic susceptibilities of clinical isolates were determined by Kirby–Bauer’s disk diffusion method. Susceptibility and resistance were determined according to the Clinical and Laboratory Standards Institute criteria [21].

Briefly, a suspension of the bacteria equal to a 0.5 McFarland standard was prepared in phosphate-buffered saline (PBS, pH 7.2) from an overnight culture. Using a swab, strains were inoculated onto a Mueller–Hinton agar (MHA; Bio-Rad, Hercules, CA, USA) plate.

Tested antimicrobials were ampicillin (10 μg), amoxicillin-clavulanic acid (20/10 μg), cefuroxime (30 μg), ceftriaxone (30 μg), ceftazidime (10 μg), ceftazidime/avibactam (30/20 μg), trimethoprim sulfamethoxazole (1.25/23.75 μg), ertapenem (10μg), imipenem (10 μg), meropenem (10 μg), gentamicin (10 μg), tobramycin (10 μg), amikacin (30 μg), ciprofloxacin (5 μg), and norfloxacin (10 µg). The susceptibility disks were purchased from Biolab Inc. (Budapest, Hungary). The plates were incubated for 16 to 18 h at 35 °C, and inhibition zones were determined visually.

### 4.4. Bacterial DNA Purification

The bacterial DNA was extracted by the QIAamp^®^ DNA Blood Mini Kit (QIAGEN Inc, Chatsworth, CA, USA) following the manufacturer’s instructions. One milliliter of log-phase culture suspension, at a concentration of 10^7^ CFU/mL, was used for the preparation. To trigger lysis of the bacterial cell wall, a preincubation step with 20 mg/mL lysozyme (in 20 mM Tris HCl, pH 8.0, 2 mM EDTA, 1.2% Triton X-100) was applied. The spin protocol was followed after incubation at 30 °C for 30 min. The final concentration of DNA was quantified using NanoDrop™ Lite spectrophotometer (Thermo Fisher Scientific™, Waltham, MA, USA) equipment. DNA samples were stored at −20 °C until further use.

### 4.5. Gene Targets

Three groups of antibiotic resistance genes were investigated in the genetic analysis. ESBL genes were *Bla*_TEM_, *Bla*_SHV_, *Bla*_OXA_, and *Bla*_CTX_; plasmid-mediated quinolone resistance genes were *qnrA*, *qnrD*, *qnrB*, *qnrS*, *oqxAB*, *aac(6′)-Ib-cr*, *qepA*, and *qnrC*. Finally, sulfonamide genes were *sul1*, *sul2*, and *sul3*.

### 4.6. Primers

Primer sets previously published in the literature were used with slight modification [22,23,24,25]. The written melting temperature (Tm) of the primers was 60 °C in all cases. However, although rare, some of the primers had higher calculated melting temperatures than published. These were modified by leaving some bases on the 5′ end of the original sequences. Thus, the specificity was unchanged but the differences in Tm were less than 1 °C. Primer sequences resulting amplicon lengths and references are listed in Table 4, Table 5 and Table 6. The published primer sequences are listed, and the modifications are underlined.

### 4.7. PCR Conditions

The same conditions and equipment were used in each PCR assay. A BIO-RAD CFX 96 instrument (Bio-Rad, Hercules, CA, USA) was used for PCR reaction. Each reaction was performed in 10 μL containing 5 μL MMX (Fermentas Probe/ROX qPCR MasterMix, Fermentas, Lithuania), 1 μL template DNA, 0.5 μM forward and reverse primers. The PCR cycling parameters were 1 cycle at 95 °C for 3 min, 40 cycles denaturation 95 °C for 15 s, annealing at 60 °C for 20 s and elongation at 72 °C for 1 min. The PCR fragments were separated by electrophoresis on 1.5% agarose gels containing GelRed Nucleic Acid Stain (10,000× in water; Biotium Inc., Hayward, CA, USA) and visualized by UV illumination (Bio-Rad Molecular Imager^®^ GelDoc™ XR+ system with ImageLab™ Software, Bio-Rad Laboratories, Inc., Hercules, CA, USA). Data were evaluated and compared with DNA ladder 100–1000 bp (Bioline, London, UK) DNA marker.

### 4.8. Bacterial RNA Purification

*E. coli* 33504 and *E. coli* 32313 strains were cultured overnight in LB broths of pH 5 to pH 8 at 37 °C with shaking (OD_600_:0.6). Bacterial suspensions were prepared with and without GEN (½ MIC) in LB medium at pH 5 to pH 8 and incubated at 37 °C with shaking. The total RNA was isolated after 8 h of culturing. RNA preparation was carried out in an RNase-free environment using NucleoSpin RNA kit (Macherey Nagel, Germany) according to the manufacturer’s instructions. Purified RNA was stored in RNase-free water in nuclease-free collection tubes and was maintained at −20 °C until quantification was performed. The concentration of the extracted RNA templates was assessed by spectrophotometry at 260 nm (Bio-Rad, Hercules, CA, USA, SmartSpec™ Plus).

### 4.9. Relative Gene Expression Analyses by Real-Time Reverse Transcriptase Quantitative Polymerase Chain Reaction (RT-qPCR)

The relative gene expression levels were determined at pH 5 to pH 8 in the presence and absence of GEN. Both *E. coli* strains were cultured in LB at pH 5 to pH 8 and total RNA was isolated after 8 h of culturing. The relative expression levels of the *marR* multiple antibiotic resistance regulator and the *sdiA* quorum sensing activator genes were determined by RT-qPCR. This involved the CFX96 Touch real-time PCR detection system (BioRad, Hercules, CA, USA), strictly following the manufacturer’s recommendations for the SensiFAST^TM^ SYBR No-ROX One-Step Kit (Bioline GmbH, Luckenwalde, Germany). Briefly, each well of the 96-well microtiter plate contained 20 µL as follows: 10 µL of the 2× SensiFAST^TM^ SYBR No-ROX One-Step Mix, 0.2 µL Reverse Transcriptase, 0.4 µL RiboSafe RNase Inhibitor, 5.4 µL Diethylpyrocarbonate (DEPC)-treated water, 500 nM of each primer and approximately 20 ng of total RNA in RNase-free water. Thermal cycling was initiated with a denaturation step of 5 min at 95 °C, followed by 40 cycles each of 10 s at 95 °C, 30 s at 57 °C, and 20 s at 72 °C. The relative quantities of the mRNA of each gene of interest were determined by ΔΔC_T_ method. Gene transcript levels were normalized against the *E. coli* housekeeping gene *GAPDH* measured in the same sample. The primers used in the assay shown in Table 7.

## 5. Conclusions

It is important to note that the colonization of bacteria depends on the characteristics of the population and density related virulence factors. It can be concluded that the pH can influence the activity of antibiotics and the function of efflux pump-related virulence factors such as quorum sensing and biofilm formation. Furthermore, the constituents and the pH of the urine can have an impact on bacterial growth. The manipulation of pH may increase the efficacy of antibiotics, especially in case of UTIs caused by multidrug resistant bacteria.

## Figures and Tables

**Table 1:** Phenotypic antibacterial susceptibility results.

Antibiotic	*E. coli* 32313	*E. coli* 33504	*K. pneumoniae* 33443	*K. pneumoniae* 33163	*P. mirabilis* 33877	*P. mirabilis* 32470
Ampicillin	22 mm S	25 mm S	6 mm R	0 mm R	0 mm R	0 mm R
AMC	22 mm S	25 mm S	23 mm S	8 mm R	28 mm S	0 mm R
Cefuroxime	22 mm S	25 mm S	20 mm S	0 mm R	28 mm S	0 mm R
Ceftriaxone	30 mm S	34 mm S	24 mm S	8 mm R	30 mm S	15 mm R
Ceftazidime	27 mm S	30 mm S	24 mm S	8 mm R	30 mm S	8 mm R
CZA	26 mm S	28 mm S	24 mm S	24 mm S	30 mm S	28 mm S
TMP/SMX	0 mm R	30 mm S	18 mm S	0 mm R	0 mm R	0 mm R
Ertapenem	35 mm S	35 mm S	30 mm S	30 mm S	30 mm S	30 mm S
Imipenem	30 mm S	30 mm S	30 mm S	30 mm S	30 mm S	30 mm S
Meropenem	30 mm S	30 mm S	30 mm S	30 mm S	30 mm S	30 mm S
Gentamicin	13 mm R	22 mm S	20 mm S	0 mm R	24 mm S	15 mm R
Tobramycin	20 mm S	22 mm S	20 mm S	15 mm I	24 mm S	14 mm R
Amikacin	20 mm S	22 mm S	20 mm S	25 mm I	24 mm S	22 mm I
Ciprofloxacin	0 mm R	30 mm S	16 mm R	0 mm R	35 mm S	0 mm R
Norfloxacin	0 mm R	30 mm S	16 mm R	0 mm R	35 mm S	0 mm R

AMC: amoxicillin-clavulanic acid, TMP/SMX: Trimethoprim Sulfamethoxazole (sumetrolim), CZA: Ceftazidime/Avibactam.

**Table 2:** ESBL, quinolone, and sulfonamide resistance genes in UTI bacterial isolates.

Resistance Type	Gene	*Escherichia coli*		*Proteus mirabilis*		*Klebsiella pneumoniae*	
		32313	33504	32470	33877	33163	33443
ESBL	*tem*	−	−	+	−	+	+
	*shv*	−	−	+	+	−	−
	*oxa*	−	−	+	−	−	−
	*ctx-m*	−	−	+	−	−	−
Fluoroquinolones	*qnrA*	−	−	−	−	−	−
	*qnrD*	−	−	−	−	−	−
	*qnrB*	−	−	−	−	−	−
	*qnrS*	−	−	−	−	−	−
	*oqxAB*	−	−	+	+	+	+
	*aac(69)-Ib-cr*	+	−	+	−	+	−
	*qepA*	−	−	−	−	−	−
	*qnrC*	−	−	−	−	−	−
Resistance type	*sul1*	−	−	−	−	−	−
	*sul2*	+	−	+	+	+	+
	*sul3*	−	−	−	+	−	−

**Table 3:** Minimal inhibitory concentrations for erythromycin, ampicillin, ciprofloxacin and gentamicin on *E. coli*, *K. pneumoniae* and *P. mirabilis* strains.

MIC (µg/mL)	Erythromycin				Ampicillin				Ciprofloxacin				Gentamicin			
	pH				pH				pH				pH			
	5	6	7	8	5	6	7	8	5	6	7	8	5	6	7	8
*E. coli* 33504	>100	>100	12.5	3.125	12.5	12.5	12.5	25	3.125	0.39	0.05	<0.05	25	6.25	1.56	0.05
*E. coli* 32313	>100	>100	>100	>100	>25	>25	>25	>25	>25	>25	25	>25	25	12.5	1.56	1.56
*K. pneumoniae* 33443	>100	>100	25	12.5	>25	>25	>25	>25	25	6.25	0.78	0.19	25	6.25	0.78	<0.05
*K. pneumoniae* 33163	>100	>100	>100	25	>25	>25	>25	>25	>25	>25	>25	12.5	>25	>25	>25	12.5
*P. mirabilis* 33877	>100	>100	>100	50	>25	>25	>25	>25	0.78	<0.05	<0.05	<0.05	>25	>25	6.25	0.39
*P. mirabilis* 32470	>100	>100	100	25	>25	>25	>25	>25	>25	>25	>25	>25	>25	>25	12.5	3.125

**Table 4:** ESBL resistance genes and primers.

Gene	Primer	Sequence (5′-3′)	Amplicon Size (bp)	Reference
*TEM*	F	CATTTCCGTGTCGCCCTTATTC	800	[22]
	R	CGTTCATCCATAGTTGCCTGAC		
*SHV*	F	AGCCGCTTGAGCAAATTAAAC	713	
	R	ATCCCGCAGATAAATCACCAC		
*OXA*	F	GGCACCAGATTCAACTTTCAAG	564	
	R	GACCCCAAGTTTCCTGTAAGTG		
*CTX-M*	F	TTTGCGATGTGCAGTACCAGTAA	544	[23]
	R	CGATATCGTTGGTGGTGCCATA		

**Table 5:** Plasmid-mediated quinolone resistance genes and primers [24].

Gene	Primer	Sequence (5′-3′)	Amplicon Size (bp)
*qnrA*	F	CAGCAAGAGGATTTCTCACG	630
	R	AATCCGGCAGCACTATTACTC	
*qnrD*	F	CGAGATCAATTTACGGGGAATA	581
	R	AACAAGCTGAAGCGCCTG	
*qnrB*	F	GGCTGTCAGTTCTATGATCG	488
	R	GAGCAACGATGCCTGGTAG	
	degR	SAKCAACGATGCCTGGTAG	
*qnrS*	F	GCAAGTTCATTGAACAGGGT	428
	R	TCTAAACCGTCGAGTTCGGCG	
*oqxAB*	F	CCGCACCGATAAATTAGTCC	313
	R	GGCGAGGTTTTGATAGTGGA	
*aac(6′)-Ib-cr*	F	TTGGAAGCGGGGACGGAM	260
	R	ACACGGCTGGACCATA	
*qepA*	F	GCAGGTCCAGCAGCGGGTAG	218
	R	CTTCCTGCCCGAGTATCGTG	
*qnrC*	F	GCAGAATTCAGGGGTGTGAT	118
	R	AACTGCTCCAAAAGCTGCTC	

**Table 6:** Sulfonamide resistance genes and primers [26].

Gene	Primer	Sequence (5′-3′)	Amplicon Size (bp)
*sul* *1*	qF	TGTCGAACCTTCAAAAGCTG	113
	qR	TGGACCCAGATCCTTTACAG	
*su* *l2*	qF	ATCTGCCAAACTCGTCGTTA	89
	qR	CAATGTGATCCATGATGTCG	
*sul* *3*	qF	GGTTGAAGATGGAGCAGATG	111
	qR	GCCTTAATGACAGGTTTGAGTC	

**Table 7:** Forward and reverse primers used for the assessment of the activity of the multiple antibiotic resistance regulator gene *marR* and the quorum-sensing regulator *sdiA* of *Escherichia coli* 33504 and 32313.

Gene	Primer	Sequence (5′-3′)	Amplicon Size (bp)	Reference
*marR*	F	AGCGATCTGTTCAATGAAAT	170	[26]
	R	TTCAGTTCAACCGGAGTAAT		
*sdiA*	F	CTGATGGCTCTGATGCGTTTA	163	[27]
	R	TCTGGTGGAAATTGACCGTATT		
*GAPDH*	F	ACTTACGAGCAGATCAAAGC	170	[26]

## Data Availability

The data presented in this study are available on request from the corresponding author.
